# Advances in Emerging and Neglected Infectious Diseases 2018

**DOI:** 10.1155/2018/4619282

**Published:** 2018-07-15

**Authors:** Charles T. Spencer, Jose R. Vasconcelos, Natalie J. Thornburg, Sara L. Zimmer

**Affiliations:** ^1^Department of Biological Sciences, University of Texas at El Paso, El Paso, TX, USA; ^2^Departamento de Biocièncias, Universidade Federal de São Paulo, Campus Baixada Santista, Santos, São Paulo, Brazil; ^3^Division of Viral Diseases, Centers for Disease Control and Prevention, Atlanta, GA, USA; ^4^Department of Biomedical Sciences, University of Minnesota Medical School, Duluth, MN, USA

Emerging and neglected infections are some of the most lethal diseases with mortality rates ranging from 10 to 80%. Despite high mortality for many of these infections, efforts at understanding them comprise an unusually small fraction of the biomedical research community's efforts. However, causative agents of emerging infections are the very ones for which an evolutionary, environmental, or nefarious alteration could allow accelerated spread amongst the human population. Furthermore, these diseases do not respect political boundaries. For example, the emerging tick-transmitted disease foci of the north central and northeastern United States of America have expanded to include parts of Canada, and recent Ebola outbreaks illustrate transmissibility across borders in the age of global economy. Thus, the international research community has a particular responsibility to direct adequate attention to studying these diseases and their etiologic agents.

Emerging and neglected infectious disease continues to be on the radar of the editors of this journal. In 2017, the journal introduced a special issue to illuminate research focused on understanding of and preparing for the emergence of these neglected diseases. The original special issue has been expanded into a continuing series. We present here the 2018 compilation of studies and a review, each addressing a specific topic within this special issue. These articles provide a snapshot of a much broader landscape of the questions and concerns of emerging and neglected infectious disease. Manuscripts published in this special issue address topics in bacterial and parasitic infections ranging from novel treatments and diagnostics to epidemiology and susceptibility. Once again, their authors span the globe, again highlighting the universality of infectious disease.

M. G. R. Muniz et al. from the United States of America demonstrate that obesity results in excessive production of leptin from adipocytes which enhance the cytokine storm following infection with* Francisella tularensis*. This heightened inflammation was associated with increased death of obese mice. This study illuminates a previously unknown risk factor for the development and severity of tularemia.

D. Kosova-Maali et al., in collaboration between France and Mexico, identified variation in two genes of* Nocardia brasiliensis* that together provide a needed method to discriminate between* Nocardia* species. The inclusion of these two genes in phylogenetic analysis can be useful for identification of species type in infected patients and enhancing diagnosis and may even have identified a new species.

V. Tique et al., in collaboration between Colombia and Cuba, analyzed clinical and epidemiological features of undifferentiated tropical febrile illness later identified as leptospirosis with the goal of refining the diagnostic features used in Colombia to identify* Leptospira *infection.

R. Holla et al. from India similarly analyzed clinical and epidemiological features of leptospirosis in coastal southern India. Accumulation of studies of the clinical and epidemiological of leptospirosis in various regions can, collectively, contribute to a better diagnosis for leptospirosis as well as reveal potential unique symptomology due to differences in the predominant strains and their microbiology.

S. C. V. Chaparro et al. from Colombia sought to improve upon the antimicrobial activity of a peptide derived from lactoferricin B. Using a polyvalent antimicrobial peptide, they demonstrate enhanced antimicrobial activity in gram-negative and gram-positive bacteria, paving the way for development of potential new therapeutics.

Y. Qi et al. from China describe a new method for the diagnosis of* Coxiella burnetti *infection resulting in Q fever. This recombinase polymerase amplification combined with lateral flow was consistent with quantitative PCR-mediated analysis but required less instrumentation. This new visual-based method is rapid and could be very beneficial in resource-limited regions.

P. M. M. Fialho et al. from Brazil present findings after following the seroconversion of* Toxocara* negative children. They identified a strong association between seroconversion and the presence of asthma. This study contributes to understanding the risk factors for seroconversion for* Toxocara* and could expand on the underlying biology of* Toxocara *disease biology.

K. Bi et al. from the United States of America present a literature review of visceral leishmaniosis prevention control strategies. They identified a lack of validation and verification between models and real-work epidemic data. Furthermore, they call for the development of more active control strategies and the advanced simulation models to predict potential pandemics.

In summary, this special issue and the work presented herein highlight the continued demand for increased understanding of infectious diseases that have received arguably less than their needed share of attention. The included studies also underscore that a lot of fundamental qualities of these diseases have yet to be addressed. For a variety of reasons, some infectious organisms and the diseases they cause will always be at the fringes of active research. It would be perilous to ignore them and this special issue is committed to throwing the spotlight on these problems in infectious disease.

*Charles T. Spencer* *Jose R. Vasconcelos* *Natalie J. Thornburg* *Sara L. Zimmer*

